# Supplementary material for: Ultrastrong coupling between nanoparticle plasmons and cavity photons at ambient conditions
Source: Nat Commun. 2020 Jun 1;11:2715. doi: 10.1038/s41467-020-16524-x (PMC7264206; doi:10.1038/s41467-020-16524-x)
Supplement: Supplementary file 1 — Supplementary Information [file 41467_2020_16524_MOESM1_ESM.pdf]

Supplementary material for

"Ultrastrong coupling between nanoparticle plasmons and cavity photons at ambient conditions"

Denis G. Baranov[1], Battulga Munkhbat[1], Elena Zhukova[2], Ankit Bisht[1], Adriana Canales[1], Benjamin Rousseaux[3], Göran Johansson[3], Tomasz Antosiewicz[1,4] and Timur Shegai[1,*]

[1]*Department of Physics, Chalmers University of Technology, 412 96, Göteborg, Sweden*

[2]*Moscow Institute of Physics and Technology, Dolgoprudny 141700, Moscow, Russia*

[3]*Department of Microtechnology and Nanoscience - MC2, Chalmers University of Technology, 412 96 Göteborg, Sweden*

[4]*Faculty of Physics, University of Warsaw, Pasteura 5, 02-093 Warsaw, Poland.*

[*]Email: timurs@chalmers.se

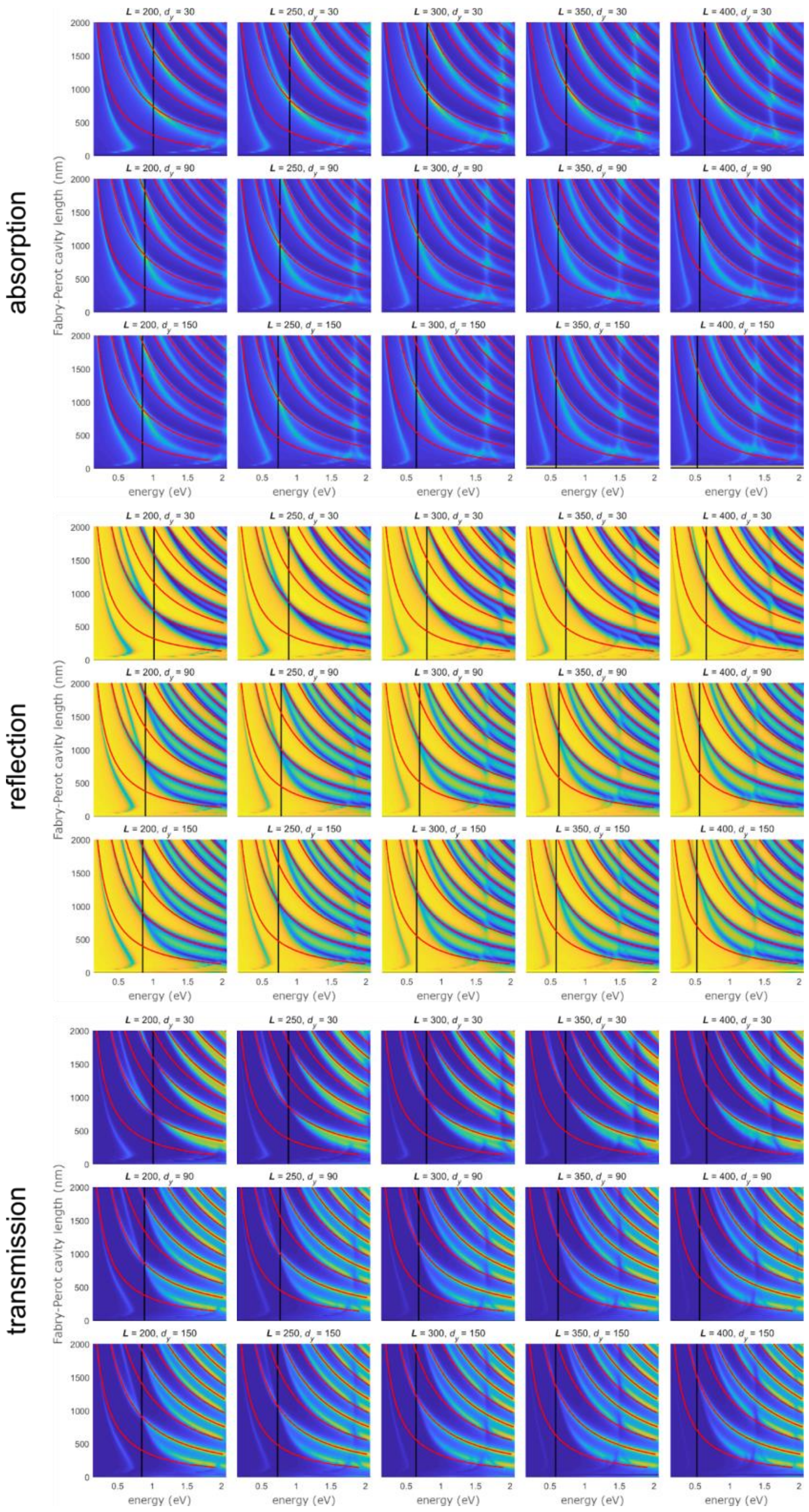

Figure S1. Calculated absorption, reflection, and transmission spectra for FP cavities with nanorod arrays for various rod lengths (L) and spacing (dy) for the electric field parallel to the nanowires. Red lines mark the FP resonances vs cavity length and the vertical black line the plasmon position of the array.

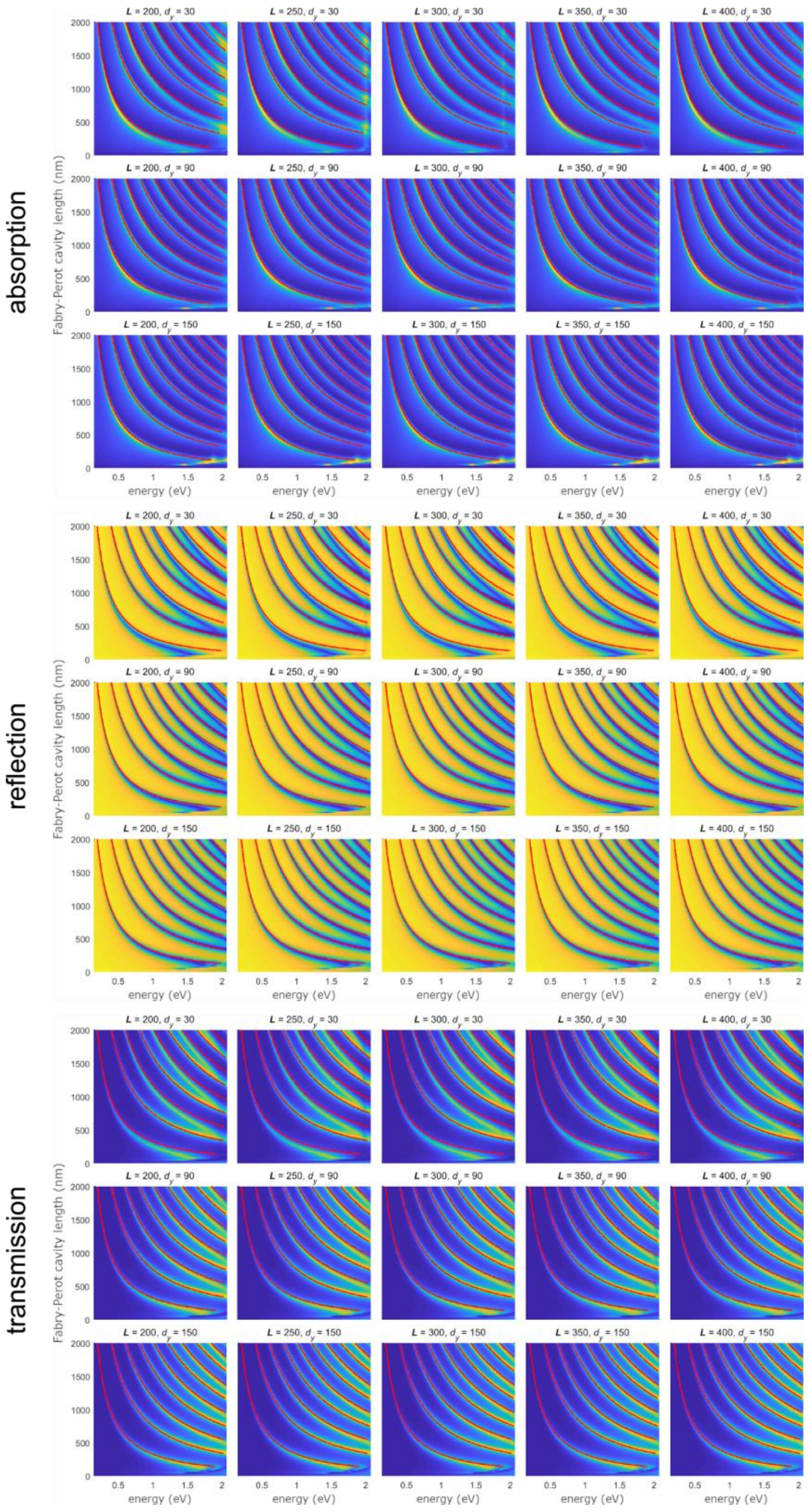

Figure S2. Calculated absorption, reflection, and transmission spectra for FP cavities with nanorod arrays for various rod lengths (*L*) and spacing (*dy*) for the electric field perpendicular to the nanowires. Red lines mark the FP resonances vs cavity length and the vertical black line the plasmon position of the array.

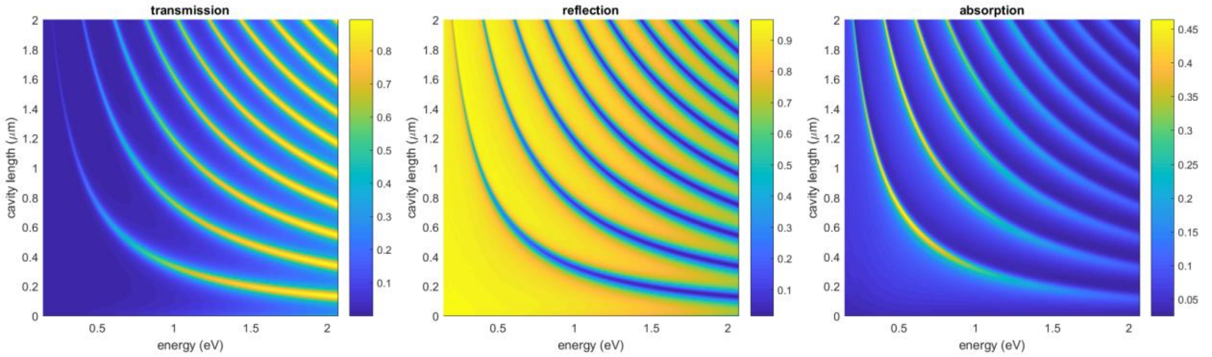

Figure S3. Calculated transmission, reflection, and absorption spectra of empty FP cavities of varied length. The subsequent resonance orders in each respective panel are traced and then plotted as reference in the spectra of total cavities.

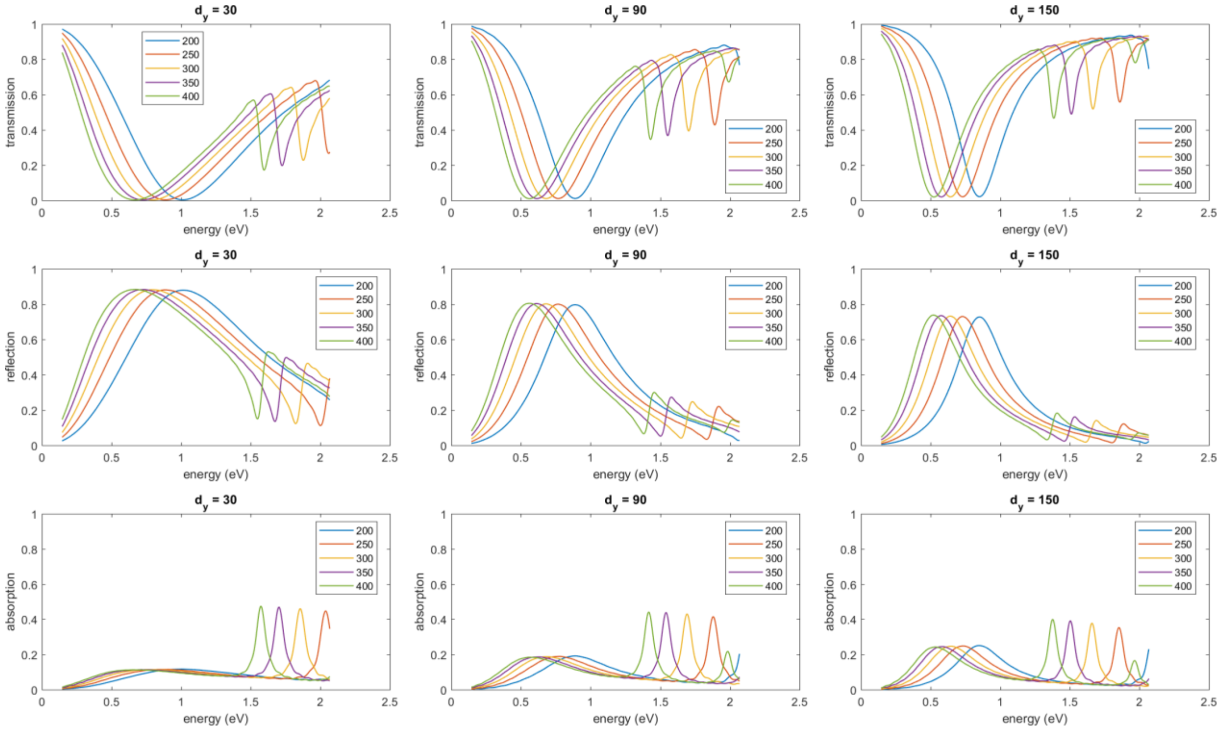

Figure S4. Calculated transmission, reflection, and absorption spectra for bare nanorod arrays with various density (*dy* = 30, 90, 150 nm) and nanorod lengths (*L* = 200-400 nm). Here polarization is parallel to the nanorods.

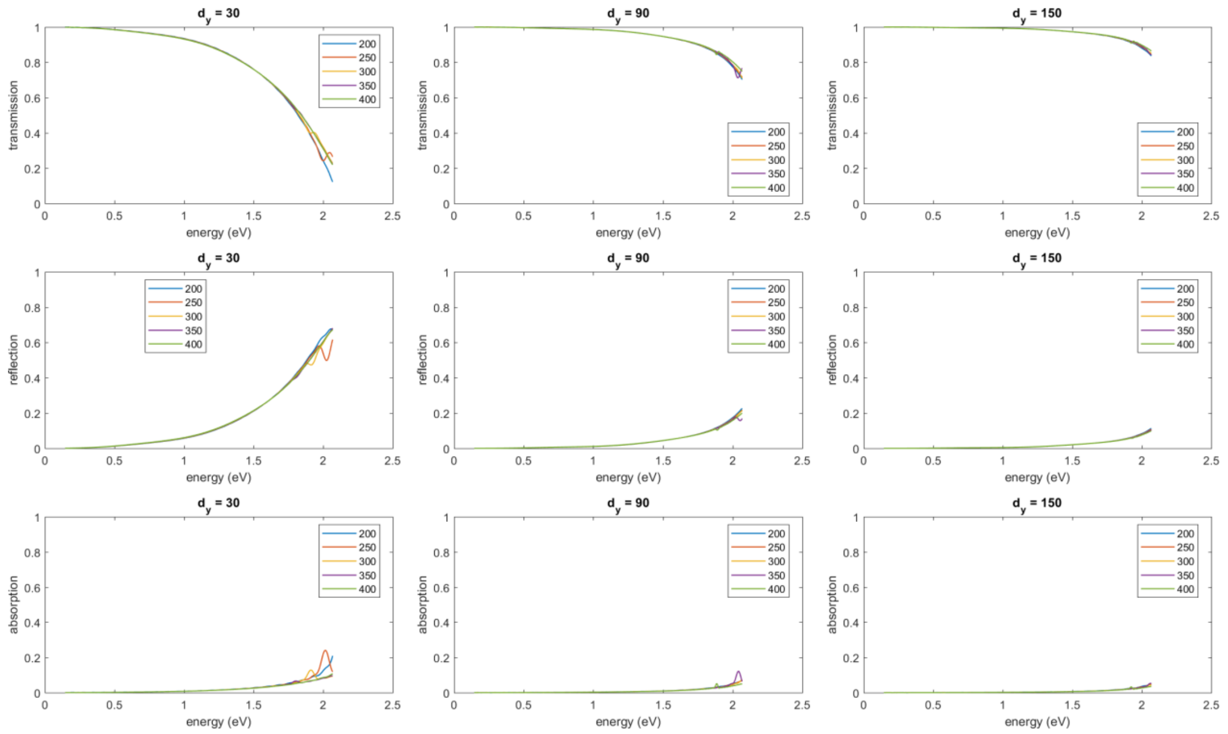

Figure S5. Transmission, reflection, and absorption spectra for bare nanorod arrays with various density (*dy* = 30, 90, 150 nm) and nanorod lengths (*L* = 200-400 nm). Here polarization is perpendicular to the nanorods.

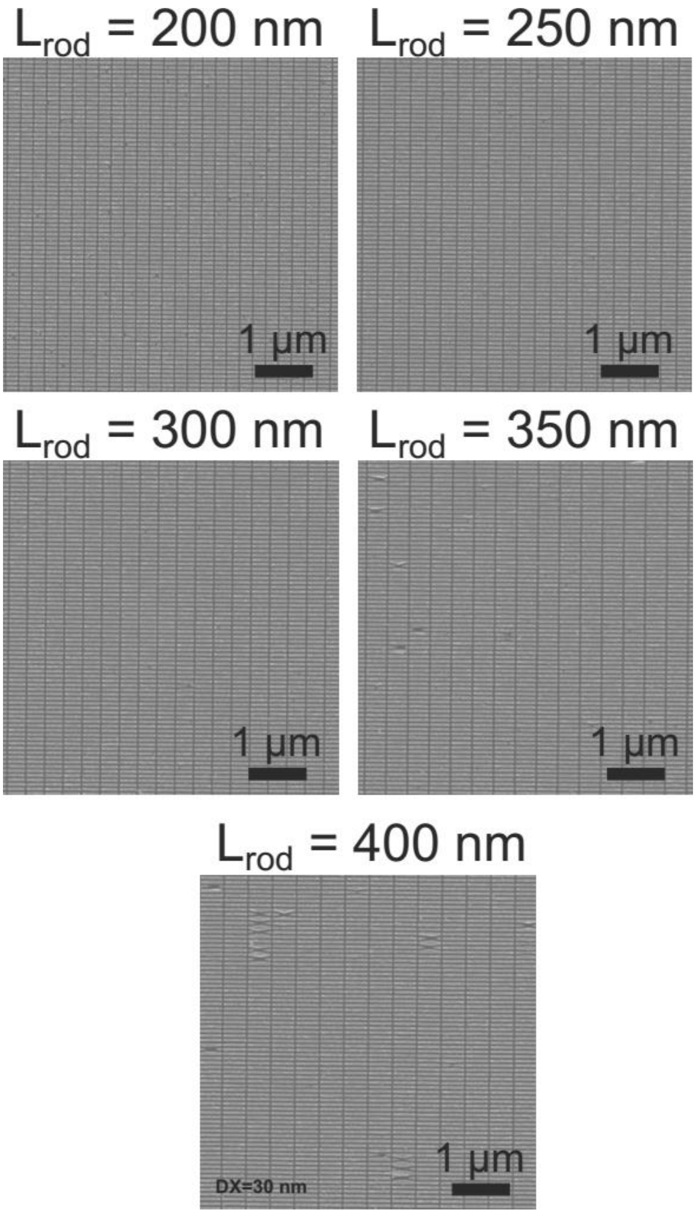

Figure S6. SEM images of the fabricated nanorod arrays with L_{rod} in the range from 200 to 400 nm.

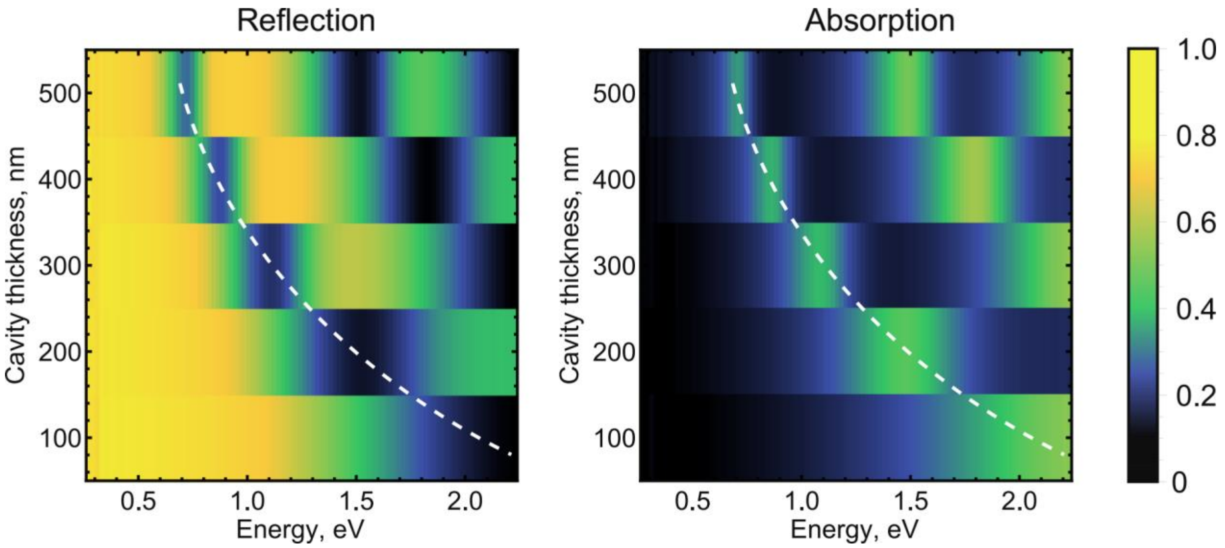

Figure S7. Experimental dispersion of normal-incidence reflection and absorption spectra of empty cavities filled with SiO_2 as a function of the cavity thickness.

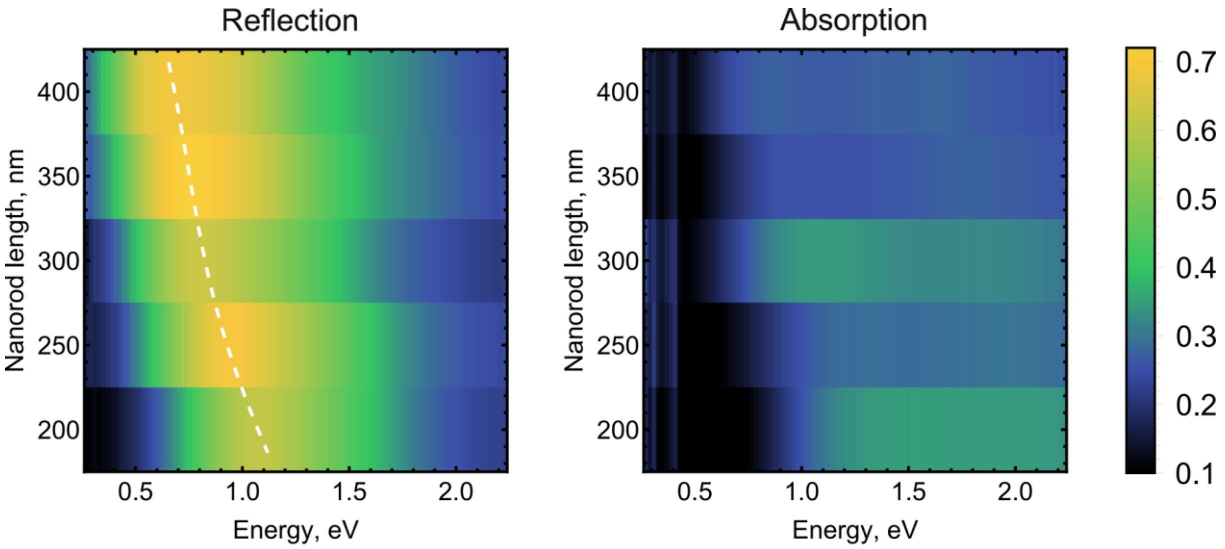

Figure S8. Experimental dispersion of normal-incidence reflection and absorption spectra of bare Au nanorod arrays fabricated on a glass substrate as a function of the nanorods length.

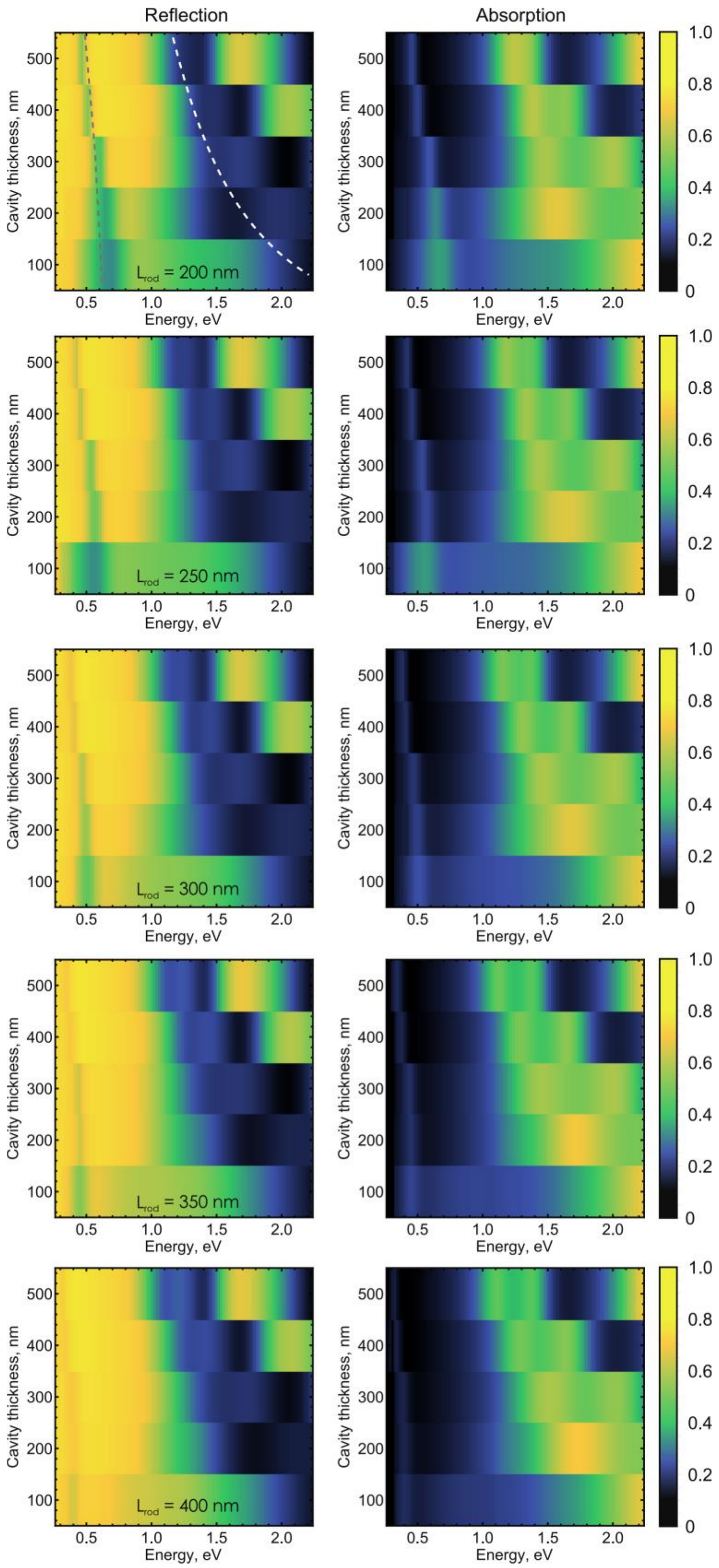

Figure S9. Experimental dispersions of the normal-incidence reflection and absorption spectra of coupled plasmon-cavity systems as a function of the cavity thickness for various nanorods length with the electric field polarization *parallel* to the nanorods axis.

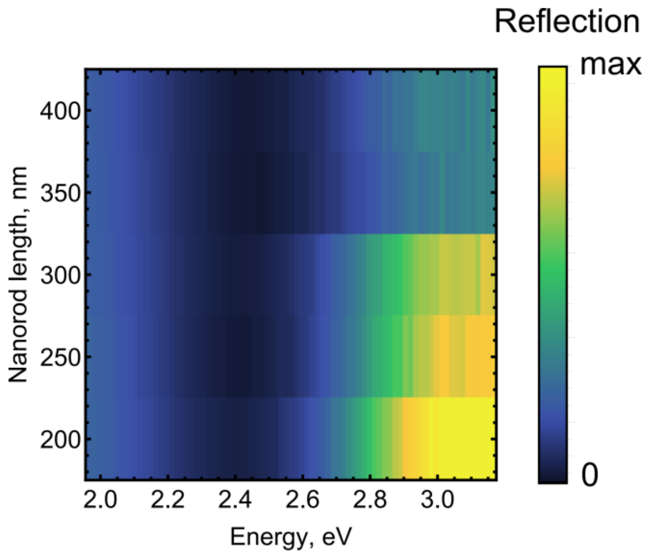

Figure S10. Experimental normal-incidence reflection spectra of coupled plasmon-cavity systems as a function nanorods length for 100 nm cavity thick samples, measured in the 2-3.3 eV range inaccessible for FTIR microscope. The reflection minima are found at around 2.35 eV, independently of the nanorod length, indicating the cavity-like character of UP states for 100 nm thick cavities.

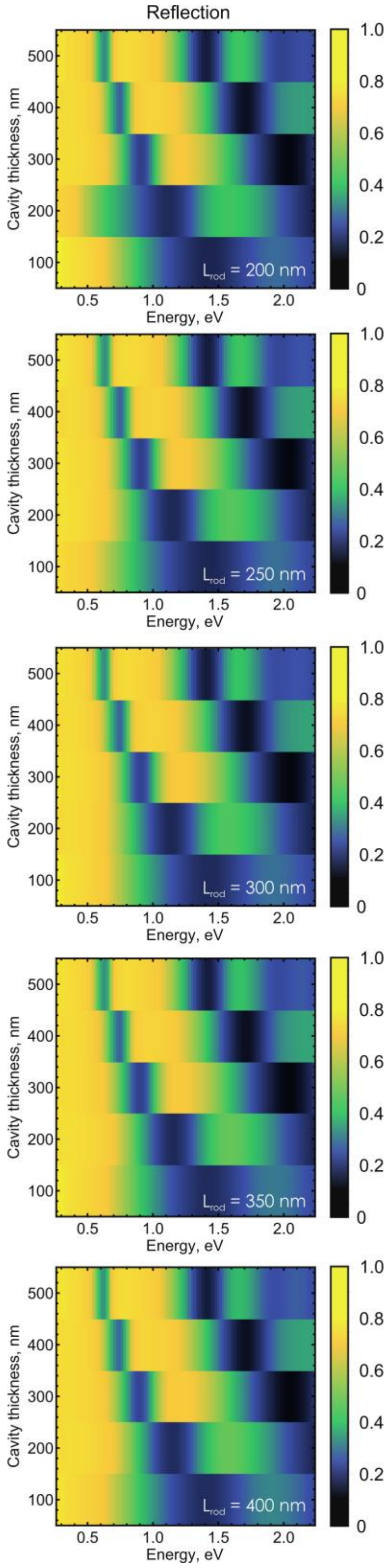

Figure S11. Experimental dispersions of the normal-incidence reflection spectra of coupled plasmon-cavity systems as a function of the cavity thickness with the electric field polarization *perpendicular* to the nanorods axis.

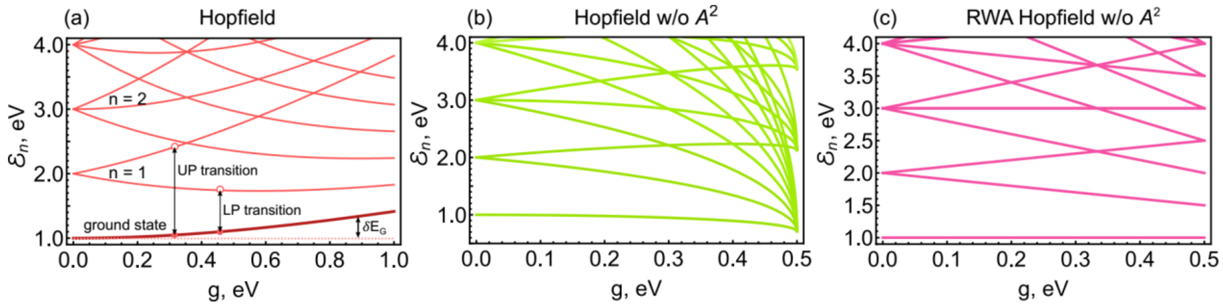

Fig. S12. (a) Eigenenergies spectrum of the Hopfield Hamiltonian (Eq. 1) plotted for $\omega_{pl} = \omega_{cav} = 1$ eV as a function of the coupling constant g. Arrows indicate resonant transitions between the ground state and two single-particle excited states that can be observed spectroscopically in a linear optical experiment. The dashed line is the constant level of 1 eV, corresponding to the ground state of the system in rotating-wave approximation. (b) Eigenenergies of Hopfield Hamiltonian without the diamagnetic A^2 term. Notice the breakdown of the energy spectrum at $g_C = \omega_{cav}/2$, associated with the square-root singularity. (c) Eigenenergies of Hopfield Hamiltonian without the diamagnetic A^2 term and the fast-rotating terms, leading to the superradiant phase transition and negative energy spectrum at $g > \omega_{cav}$.

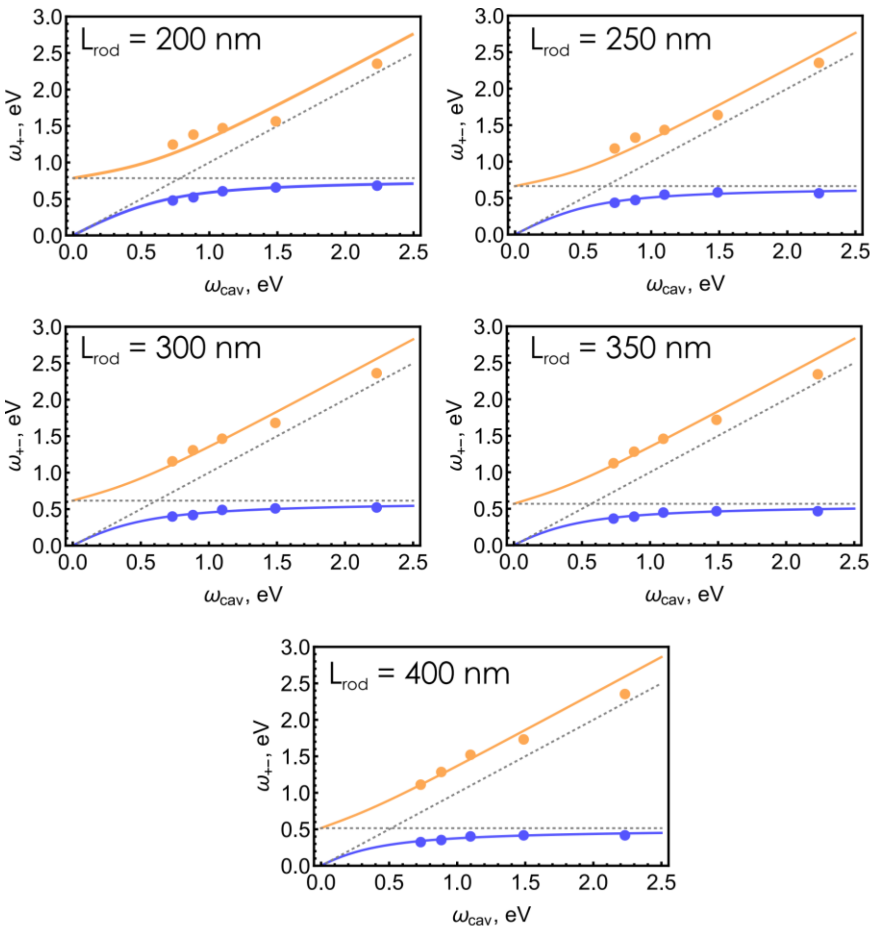

Figure S13. Fitting of the measured polaritonic dispersions of the coupled plasmon-cavity system with the single-mode Hopfield Hamiltonian transition energies for all nanorod lengths ranging from 200 to 400 nm. Circles show resonant energies of the coupled system extracted as reflection dips, lines are Hopfield polaritons dispersion, gray dashed lines are the bare cavity and bare plasmon energies.

L_{rod}, nm	200	250	300	350	400
ω_{pl}, eV	0.83	0.71	0.64	0.58	0.52
g_C, eV	0.33	0.31	0.3	0.3	0.29

Table SI. The values of bare plasmon energies and Coulomb gauge coupling strengths obtained by fitting the polariton dispersion by eigenvalues of the single-mode Hopfield Hamiltonian.

Note S1. Multi-mode Hopfield Hamiltonian.

To describe coupling of the nanorod array with all Fabry-Pérot modes of the cavity, we adopt the multi-mode Hopfield Hamiltonian in the Coulomb gauges from [1]:

$$\hat{H} = \sum_j \hbar\omega_{cav}^{(j)}\left(\frac{1}{2} + \hat{a}_j^\dagger \hat{a}_j\right) + \hbar\omega_{pl}\left(\frac{1}{2} + \hat{b}^\dagger \hat{b}\right) + \hat{H}_{int},$$

$$\hat{H}_{int} = \sum_j \hbar g_C^{(j)}\left(\hat{a}_j^\dagger + \hat{a}_j\right)\left(\hat{b}^\dagger + \hat{b}\right) + \sum_{j,k} \frac{\hbar g_C^{(j)} g_C^{(k)}}{\omega_{pl}}\left(\hat{a}_j^\dagger + \hat{a}_j\right)\left(\hat{a}_k^\dagger + \hat{a}_k\right),$$

where $\hat{a}_j$ is the annihilation operator of the j-th FP mode having the resonant energy $\omega_{cav}^{(j)}$, and $g_C^{(j)}$ is the coupling constant with j-th FP mode. We will assume here that the cavity is formed by perfect electric conductor mirrors; hence, at normal incidence ($k_{\parallel} = 0$), the energy of the j-th cavity mode is $\omega_{cav}^{(j)} = j\omega_{cav}$, and the coupling constant in the Coulomb gauge scales as $g_C^{(j)} = g_C/\sqrt{j}$ with g_C being the coupling constant to the fundamental FP mode. The spectrum of this Hamiltonian $\{\omega\}$ is given by non-negative roots of the following equation [2]:

$$\omega_{pl}^2 - \omega^2 = \frac{2\pi g_C^2 \omega}{\omega_{pl}} \frac{\sin^2 \dfrac{\pi\omega}{2\omega_{cav}}}{\sin \dfrac{\pi\omega}{\omega_{cav}}}.$$

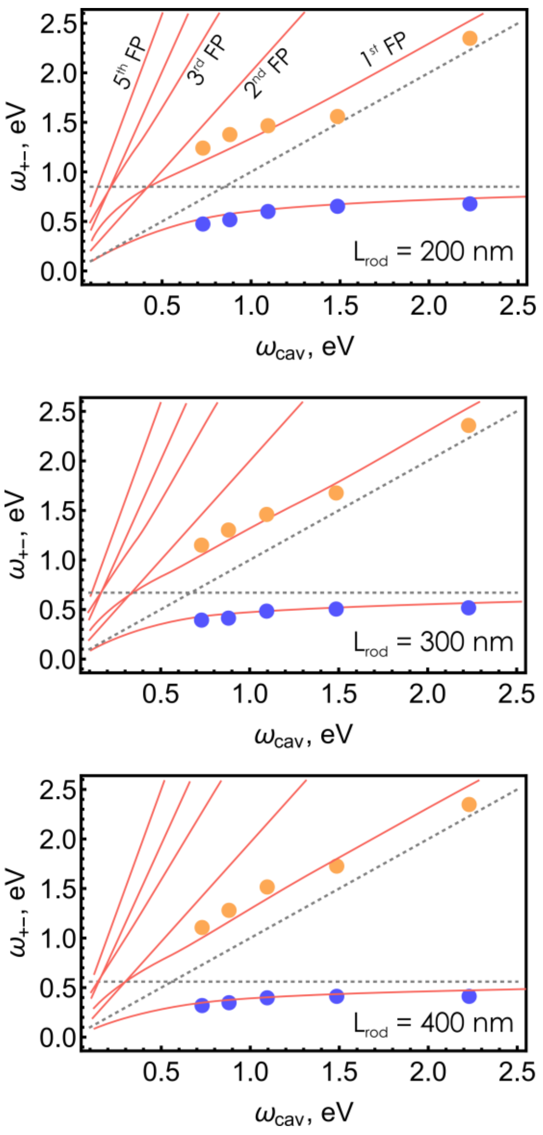

Figure S14. Fitting of the experimental polariton dispersions with the multimode normal-incidence Hopfield Hamiltonian.

L_{rod}, nm	200	300	400
ω_{pl}, eV	0.85	0.67	0.56
g_C, eV	0.35	0.33	0.32

Table SII. The values of bare plasmon energies and Coulomb gauge coupling strengths obtained by fitting the polariton dispersion with eigenvalues of the multimode Hopfield Hamiltonian.

Note S2. Transition dipole moment of a single plasmonic nanorod

In order to estimate the effective transition dipole moment of a plasmonic nanorod μ, in analogy to a two-level system, we employ the coupled-mode theory for an arbitrary three-dimensional scatterer in free space [3]. The scattering cross-section on resonance reads:

$$\sigma_{scat} = (2\ell + 1)\lambda^2 \left| \frac{\gamma_{rad}}{\gamma_{rad} + \gamma_{n-rad}} \right|^2,$$

where γ_{rad} and γ_{n-rad} are the radiative and non-radiative decay rates of the plasmon mode, and $\ell = 1$ for the dipolar scattering channel. Thus, by extracting the total plasmon decay rate $\gamma_{rad} + \gamma_{n-rad}$ as the full width of the scattering peak and the resonant value of the scattering cross-section (Fig. S12), we are able to calculate its pure radiative decay rate. Next, we estimate the transition dipole moment by using the Larmor formula for the radiative decay rate of an electric dipole $\gamma_{rad} = \frac{\omega_0^3}{3\pi\hbar\varepsilon_0 c^3}|\mu|^2$ [4], yielding around 34 000 D for the 400 nm long Au nanorod.

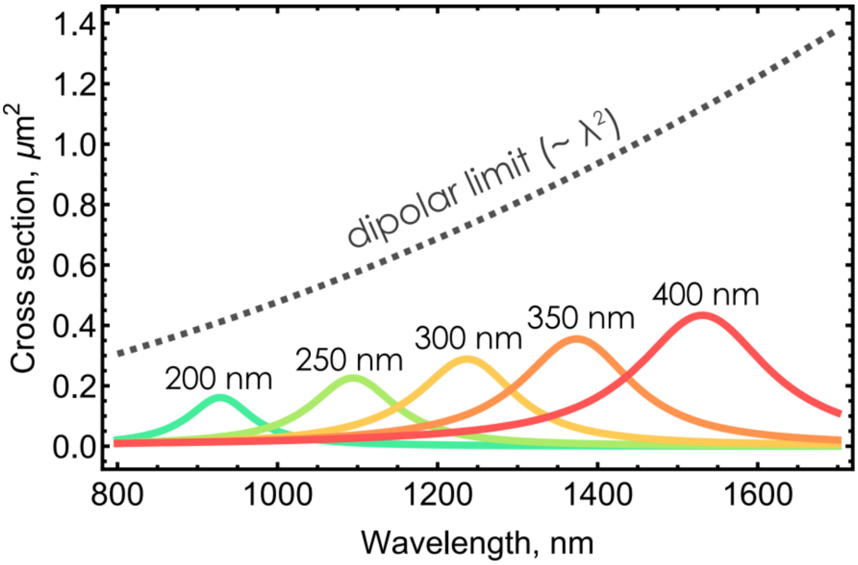

Figure S15. Scattering cross-section of single Au nanorods of various lengths (height $h = 20$ nm, width $w = 50$ nm corresponding to the analyzed arrays) in air calculated with the finite-difference time-domain solver (Lumerical), and the dipolar limit $\sigma_0 = \frac{3}{2\pi}\lambda^2$ for the scattering cross-section imposed by the energy conservation.

L_{rod}, nm	200	250	300	350	400
ω_{pl}, eV	1.33	1.13	1.0	0.9	0.81
γ_{tot}, meV	140	134	122	115	107
γ_{rad}, meV	88	84	76	73	67
μ, 10^3 D	18.5	23	26.3	30.2	33.8

Table SIII. Resonant frequencies, total decay rates, radiative decay rates, and the effective transition dipole moments of single Au nanorods of different lengths estimated from finite difference time domain simulations.

Similarly, to estimate the radiative decay rate of a nanorod array, we simulate the normal incidence reflection spectrum of the array illuminated with a plane wave. Reflection of a single-mode system at resonance can be well approximated by $\left|\frac{\gamma_{rad}}{\gamma_{tot}}\right|^2$ [5,6]. Therefore, for the 400 nm long nanorods array with 30 nm spacing we obtain $\gamma_{rad}/\gamma_{tot}$~0.95, indicating that the radiative decay of the plasmonic array is the dominant decay process.

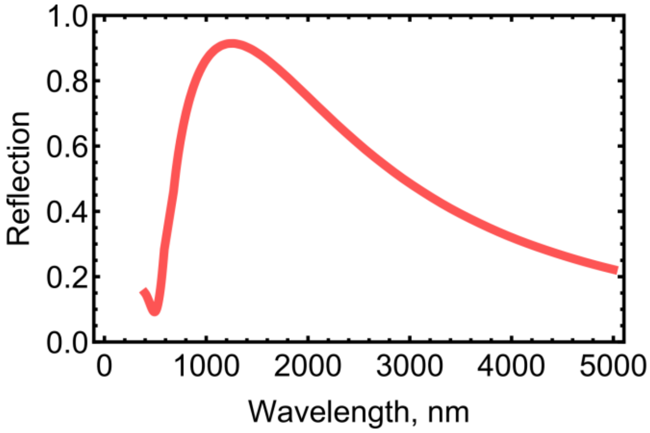

Figure S16. Reflection spectrum of a 400 nm long nanorods array in air calculated with the finite-difference time-domain solver (Lumerical).

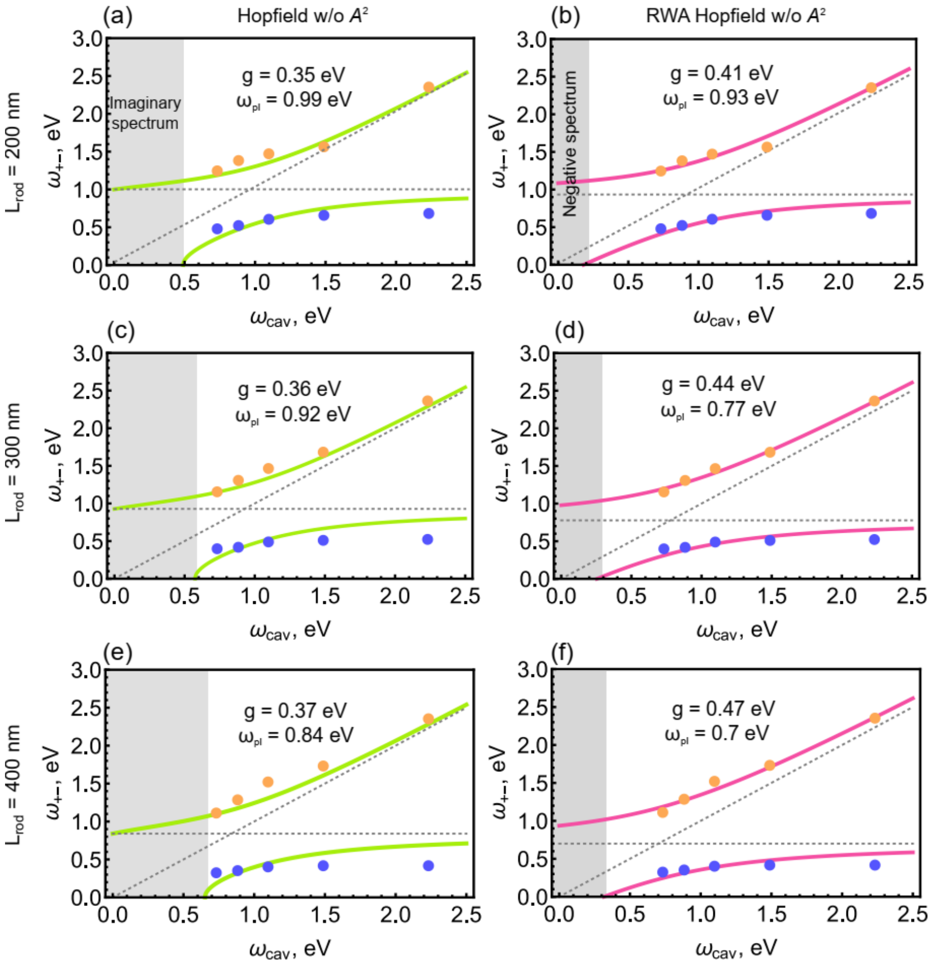

Figure S17. (a, c, e) Fitting of the measured polaritonic dispersions of the coupled systems with $L_{rod} = 200$, 300, and 400 nm with transition energies of the single-mode Hopfield Hamiltonian without the A^2 term. (b, d, f) Fitting the same data with Hopfield Hamiltonian under rotating wave approximation and no A^2 term.

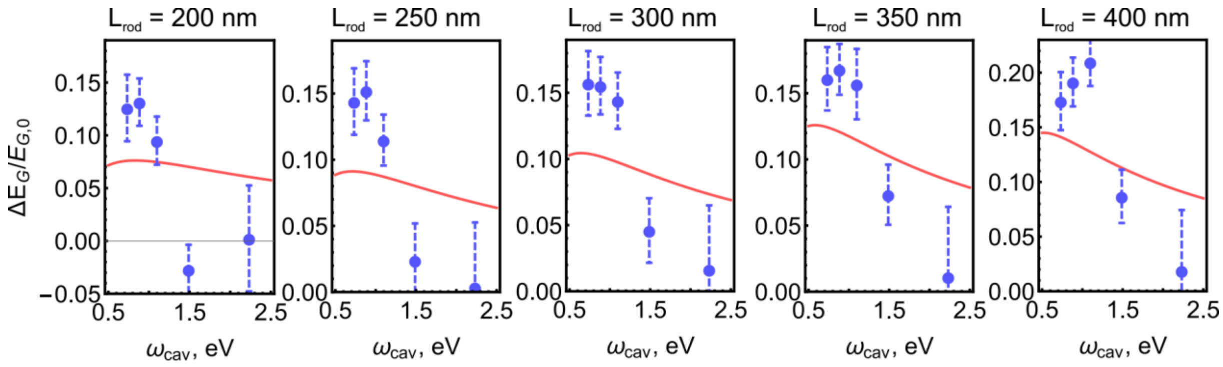

Figure S18. The normalized vacuum energy variation $\delta E_G/E_G = \frac{\tilde{E}_G - E_G}{E_G}$ of the coupled plasmon-cavity system as a function of the bare cavity energy for normal incidence eigenmodes calculated with the coupling strengths obtained from the single-mode Hamiltonian fitting for various nanorod lengths (curves), as well as the vacuum energy variation calculated directly from the measured polariton energies (circles).

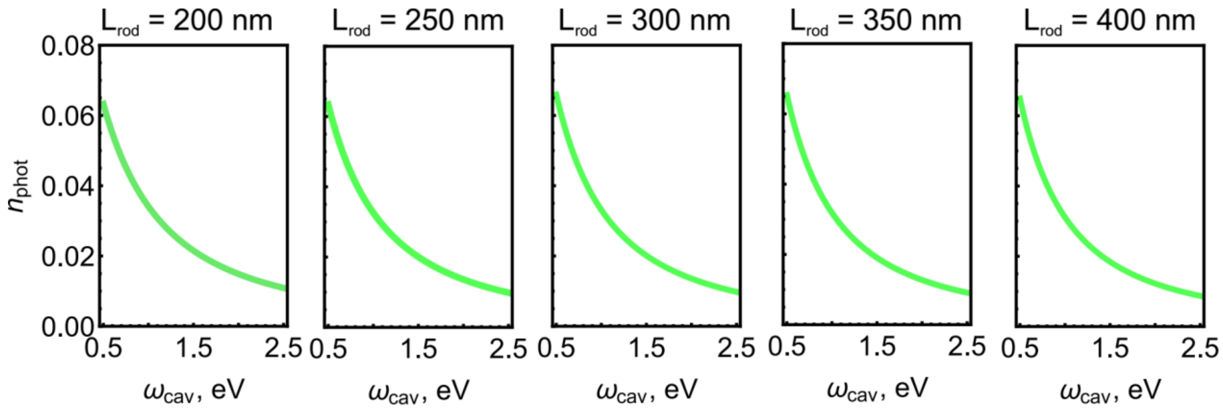

Figure S19. The photonic occupancy of the modified ground state $\tilde{n}_{phot} = \langle \tilde{G}|\hat{a}^{\dagger}\hat{a}|\tilde{G}\rangle$ of coupled plasmon-cavity systems as a function of the bare cavity energy calculated with the coupling strengths obtained from the single-mode Hamiltonian fitting for various nanorod lengths.

[1] D. De Bernardis, P. Pilar, T. Jaako, S. De Liberato, and P. Rabl, *Breakdown of gauge invariance in ultrastrong-coupling cavity QED*, Phys. Rev. A **98**, 053819 (2018).

[2] S. De Liberato, *Light-matter decoupling in the deep strong coupling regime: The breakdown of the purcell effect*, Phys. Rev. Lett. **112**, 016401 (2014).

[3] R. E. Hamam, A. Karalis, J. D. Joannopoulos, and M. Soljačić, *Coupled-mode theory for general free-space resonant scattering of waves*, Phys. Rev. A - At. Mol. Opt. Phys. **75**, 1 (2007).

[4] L. Novotny and B. Hecht, *Principles of Nano-Optics* (Cambridge University Press, 2006).

[5] S. Fan, W. Suh, and J. D. Joannopoulos, *Temporal coupled-mode theory for the Fano resonance in optical resonators*, J. Opt. Soc. Am. A **20**, 569 (2003).

[6] S. Huppert, A. Vasanelli, G. Pegolotti, Y. Todorov, and C. Sirtori, *Strong and ultrastrong coupling with free-space radiation*, Phys. Rev. B **94**, 155418 (2016).
